# Cryo Electron Tomography of Native HIV-1 Budding Sites

**DOI:** 10.1371/journal.ppat.1001173

**Published:** 2010-11-24

**Authors:** Lars-Anders Carlson, Alex de Marco, Heike Oberwinkler, Anja Habermann, John A. G. Briggs, Hans-Georg Kräusslich, Kay Grünewald

**Affiliations:** 1 Department of Infectious Diseases, Virology, Universitätsklinikum Heidelberg, Heidelberg, Germany; 2 Department of Molecular Structural Biology, Max-Planck-Institute of Biochemistry, Martinsried, Germany; 3 Structural and Computational Biology Unit, European Molecular Biology Laboratory, Heidelberg, Germany; 4 Oxford Particle Imaging Centre, Division of Structural Biology, Wellcome Trust Centre for Human Genetics, University of Oxford, Oxford, United Kingdom; Northwestern University, United States of America

## Abstract

The structure of immature and mature HIV-1 particles has been analyzed in detail by cryo electron microscopy, while no such studies have been reported for cellular HIV-1 budding sites. Here, we established a system for studying HIV-1 virus-like particle assembly and release by cryo electron tomography of intact human cells. The lattice of the structural Gag protein in budding sites was indistinguishable from that of the released immature virion, suggesting that its organization is determined at the assembly site without major subsequent rearrangements. Besides the immature lattice, a previously not described Gag lattice was detected in some budding sites and released particles; this lattice was found at high frequencies in a subset of infected T-cells. It displays the same hexagonal symmetry and spacing in the MA-CA layer as the immature lattice, but lacks density corresponding to NC-RNA-p6. Buds and released particles carrying this lattice consistently lacked the viral ribonucleoprotein complex, suggesting that they correspond to aberrant products due to premature proteolytic activation. We hypothesize that cellular and/or viral factors normally control the onset of proteolytic maturation during assembly and release, and that this control has been lost in a subset of infected T-cells leading to formation of aberrant particles.

## Author Summary

The production of new HIV-1 particles is initiated at the plasma membrane where the viral polyprotein Gag assembles into a budding site, and proceeds through release of an immature virion which is subsequently transformed to the infectious virion by proteolytic cleavage of Gag. Here, we established experimental systems to study HIV-1 budding sites by cryo electron tomography. This technique allows three-dimensional structure determination of single objects at macromolecular resolution, thus being uniquely suited to study variable structures such as HIV-1 particles and budding sites. Using cryo electron tomography, we obtained three-dimensional images with unprecedented detail of the formation of HIV-1 particles. By analyzing these images we show that the organization of released immature HIV-1 is determined at its intracellular assembly without major subsequent rearrangements. We further identify a lattice structure of the viral protein Gag present in budding sites that seem to lack the viral genome and thus cannot be precursors of infectious viruses. We show that some HIV-1 infected T-cells preferentially carry these budding sites, suggesting that they have lost a crucial control of the proteolytic maturation of the virus.

## Introduction

HIV-1 particles are assembled at the cell membrane, as the 55 kDa viral polyprotein Gag multimerizes on its inner face [1]. Gag recruits other viral components such as the RNA genome and the surface spike proteins, as well as cellular proteins of the ESCRT machinery required for virus release [2], [3], [4]. The viral protease (PR) is essential to convert the immature form of the virion into an infectious mature particle. Both forms of the virion are pleiomorphic structures, with the repetitive structural elements of the virus arranged non-symmetrically and variably from one particle to the other.

In the immature virion, uncleaved Gag is anchored to the plasma membrane via a charged surface and a myristoyl tail in its N-terminal matrix (MA) domain [1]. As shown by cryo electron microscopy (cEM), Gag arranges in a regular manner, with its internal capsid (CA) domain forming a hexameric lattice with a spacing of 8.0 nm [5]. C-terminally of CA, the nucleocapsid (NC) domain binds the RNA genome, and the p6 domain recruits the ESCRT machinery to facilitate particle release [6], [7]. CA and NC, as well as NC and p6, are separated by short spacer peptides (SP1 and SP2, respectively) which are processed during maturation.

Proteolytic maturation of HIV-1 has been proposed to initiate at or shortly after assembly and release [8]. The active dimeric form of the viral PR cleaves Gag and GagPol at multiple sites, leading to the structural transition from the immature particle with its Gag shell forming a truncated sphere to the mature particle with its cone-shaped CA core encasing the condensed nucleoprotein complex in the interior of the virion. In an *in vitro* study Pettit *et al*. detected large differences in processing kinetics at the five cleavage sites in Gag, the cleavage at the site between SP1 and NC being an order of magnitude faster than the second fastest cleavage event [9]. These results suggested ordered processing during virion maturation, and this was supported by mutagenesis studies of individual cleavage sites [10]. Processing at all sites except NC-SP1 [11] appears to be important for infectivity and small amounts of partially processed Gag products have been shown to exhibit a strong *trans*-dominant negative effect on viral infectivity [12], [13], [14]. Furthermore, premature processing [15] as well as low concentrations of protease inhibitors, insufficient to significantly affect proteolytic maturation [14], [16], [17], were both shown to efficiently block viral infectivity, indicating an intricate interplay between proteolytic activation and virus formation. Morphological maturation is believed to occur rapidly following release of the immature virion as no intermediates of maturation have been observed so far and all cellular budding sites appear to carry an immature Gag shell.

The last years have seen increasingly detailed structural studies of released HIV-1 particles in their immature and mature forms [18], [19], [20], [21], [22], [23], [24], [25]. Analysis of immature virions by cryo electron tomography (cET) revealed that the immature lattice covers only part of the viral membrane [22], arranging as an incomplete “∼2/3” sphere [26]. These studies suggested that ESCRT involvement in HIV-1 release occurs earlier than previously thought and that so-called late budding sites carrying an almost complete Gag lattice are likely to be dead-end products rather than intermediates in the release pathway [26]. A later analysis by cET and subtomogram averaging provided a 17 Å structure of the immature Gag lattice and showed how this incomplete hexagonal lattice attains its curvature through inclusion of symmetry defects of irregular shape and size [19]. In the mature capsid, CA also forms a hexameric lattice, but with a different lattice constant and arrangement than in the immature lattice [27]. Curvature of the mature capsid is achieved by asymmetric incorporation of pentameric defects at the narrow and wide end of the cone [28]. While cET studies of released virions have significantly advanced our understanding of HIV-1 morphogenesis, we are currently lacking any three-dimensional information regarding native intracellular particle assembly and budding. These processes have hitherto been inaccessible for detailed structural studies due to the lack of an experimental system enabling their visualization by cET. Here, we present a structural study of HIV-1 assembly sites by cET of intact, plunge-frozen human cells. These snapshots allow detailed structural interpretations of the Gag lattice structure at its assembly site *in situ*, as well as the arrangement of the cortical actin in its immediate vicinity. We further determined the structure of a previously not reported Gag lattice type lacking the NC-RNA layer which we propose to be associated with loss of control of PR activation.

## Results

### Visualization of HIV-1 assembly by cryo electron tomography

**Figure 1:**
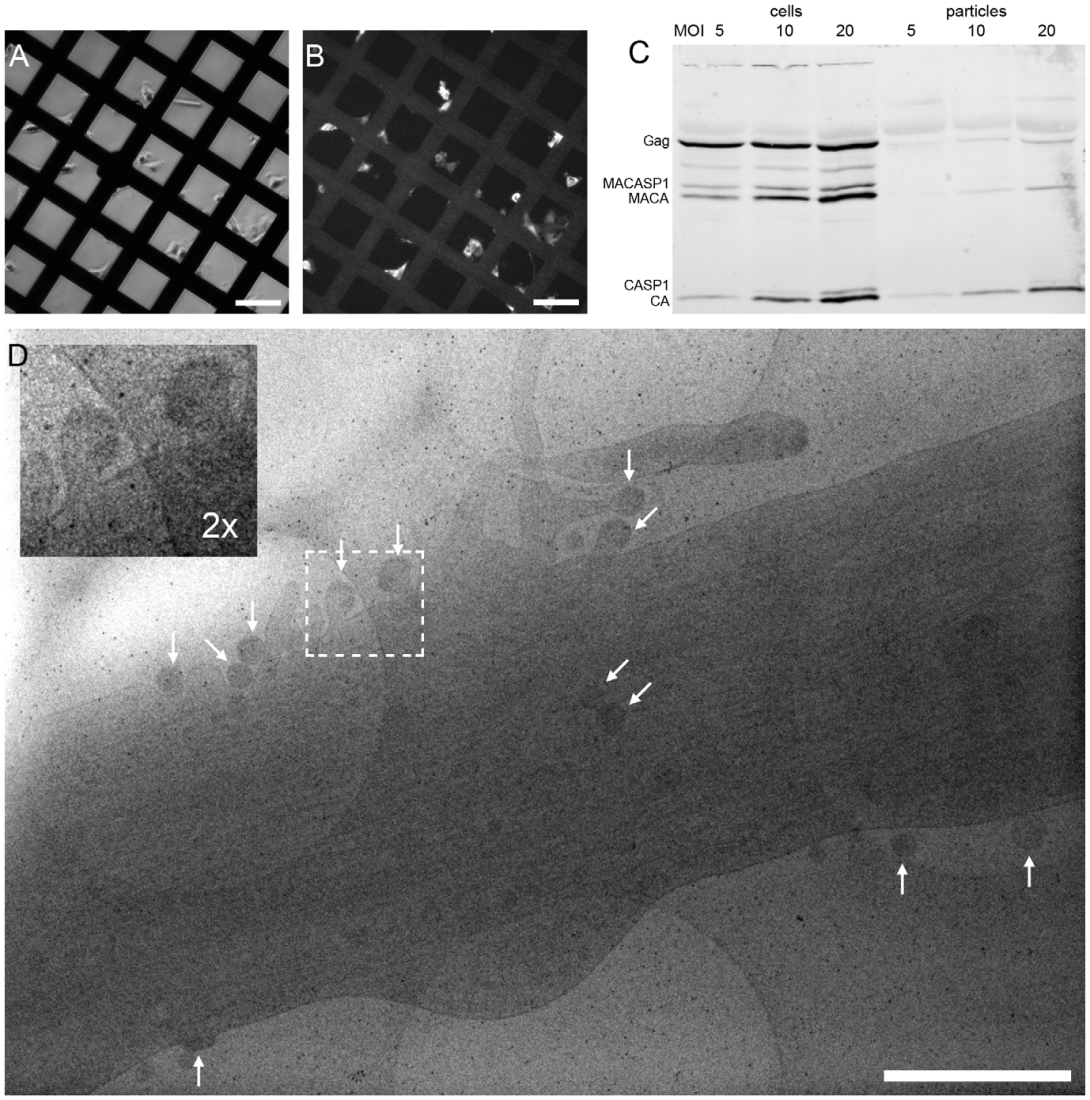
HIV-1 assembly sites imaged by cryo electron microscopy of intact human cells. (A) Light microscopy phase contrast and (B) immunofluorescence image of human glioblastoma cells (U-373 MG) grown adherently on carbon coated EM grids. Cells were transduced with AdGag concomitant with seeding on grids, and subjected to immunofluorescence staining for Gag two days post seeding to estimate the relative number of Gag expressing cells. (C) U373-MG cells were transduced with AdGagPol at various MOI as indicated above the panel. Cell and particle lysates were recovered 2 days after transduction and subjected to immunoblot analysis using polyclonal antiserum raised against CA. The positions of the Gag and GagPol polyproteins and of several processing products are indicated on the left. (D) Cryo electron micrograph (projection image) of a U-373 MG cell plunge-frozen two days after transduction with AdGagPol. The image shows a cell protrusion containing viral assembly sites (marked by arrows) accessible for cET (The large light circles are 2 µm diameter holes in the carbon support films that the cells grow on, the small dark spots are 10 nm colloidal gold beads). The inset in the upper left corner is a magnified view of the boxed area in the large image. Scale bars are 100 µm (A–B) and 1 µm (D).

To enable structural analysis of HIV-1 assembly in its cellular context, we set out to establish a system for studying this process by cET on intact human cells. Since cET is limited to samples thinner than ∼500 nm, and thus to thin peripheral areas of adherent cells, the natural HIV-1 host cell types (T-cells and macrophages) cannot be used for cET. This led us to use two human glioblastoma cell lines, U-87 MG and U-373 MG. These cells grow adherently, have extended, thin peripheral areas, and are amenable to transfection or transduction with adenoviral vectors. Transfection efficiency proved insufficient for structural analyses, however, and we therefore constructed adenoviral vectors expressing HIV-1 Gag (AdGag) and HIV-1 Gag-Pol (AdGagPol), respectively. Both adenoviral vectors were based on Rev-independent versions of the respective genes [29], allowing expression of either Gag or Gag-Pol without co-transduction of Rev. U-87 MG or U-373 MG cells were seeded on EM sample grids concomitant with transduction using the respective adenoviral vector. Immunofluorescence staining for HIV-1 CA at 24–48 h post transduction revealed that almost 100% of the cells expressed HIV-1 Gag, while retaining normal adherent morphology (Fig. 1A–B). Furthermore, when U-373 MG cells were transduced with AdGagPol, specific cleavage products of HIV-1 Gag were found in the cell lysate and in the particle fraction purified from the culture medium (Fig. 1C), while particle release but no processing was observed for AdGag (data not shown). Cells transduced with AdGag or AdGagPol were vitrified by plunge-freezing and subjected to cEM. Upon inspection of the vitrified grids, HIV-1 assembly sites and released virions were present at low but reproducible frequencies at the periphery of the cells (Fig. 1D) in regions accessible to cET. A total of 40 tilt series were recorded on such positions of AdGag and AdGagPol infected cells, respectively (Table 1).

**Table 1:** Data statistics for cET of HIV-1 budding.

Data set number	Sample		Number of budding sites	Number of released particles
1	U-87 MG transduced with AdGag	6	4	5
2	U-373 MG transduced with AdGag	9	9	26
3	U-373 MG transduced with AdGagPol	25	26	39

For each data set, the number of recorded tilt series is stated along with the number of budding sites and released particles contained in the data set.

**Figure 2:**
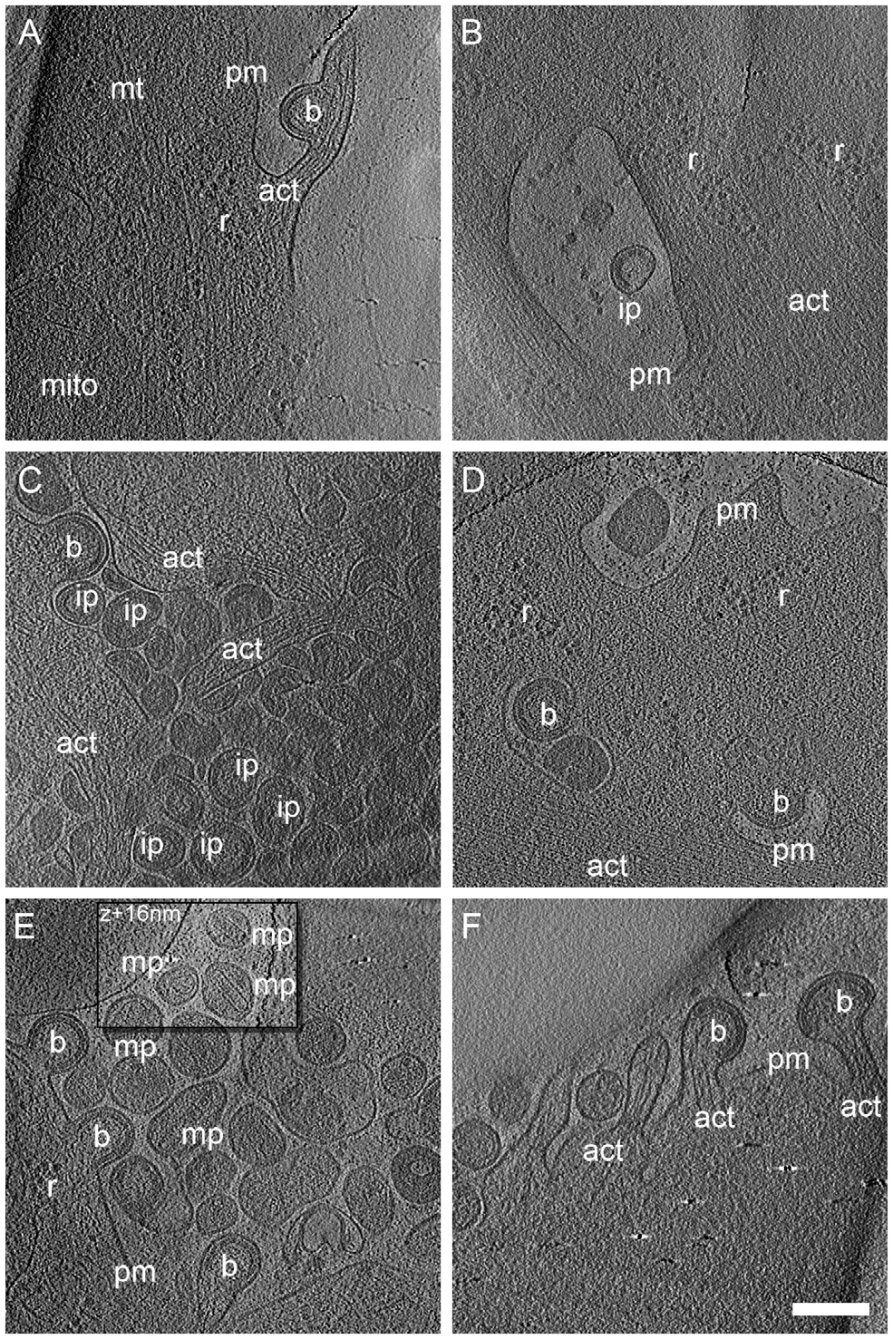
Cryo electron tomograms of HIV-1 budding sites and released particles. Computational slices of 1.6 nm thickness through cryo electron tomograms of U-87 MG (A–B) and U-373 MG (C–F) cells transduced with AdGag (A–C) or AdGagPol (D–F). Density is black. *Bona fide* designations of structures: act, actin; b, budding sites; ip, immature particles; mp, mature particles; mt, microtubule; r, ribosome; pm, plasma membrane. The inset in (E) is shifted by 16 nm perpendicular to the image plane, at which location the morphology of the mature particles appears clearer. Scale bar is 200 nm.

**Figure 3:**
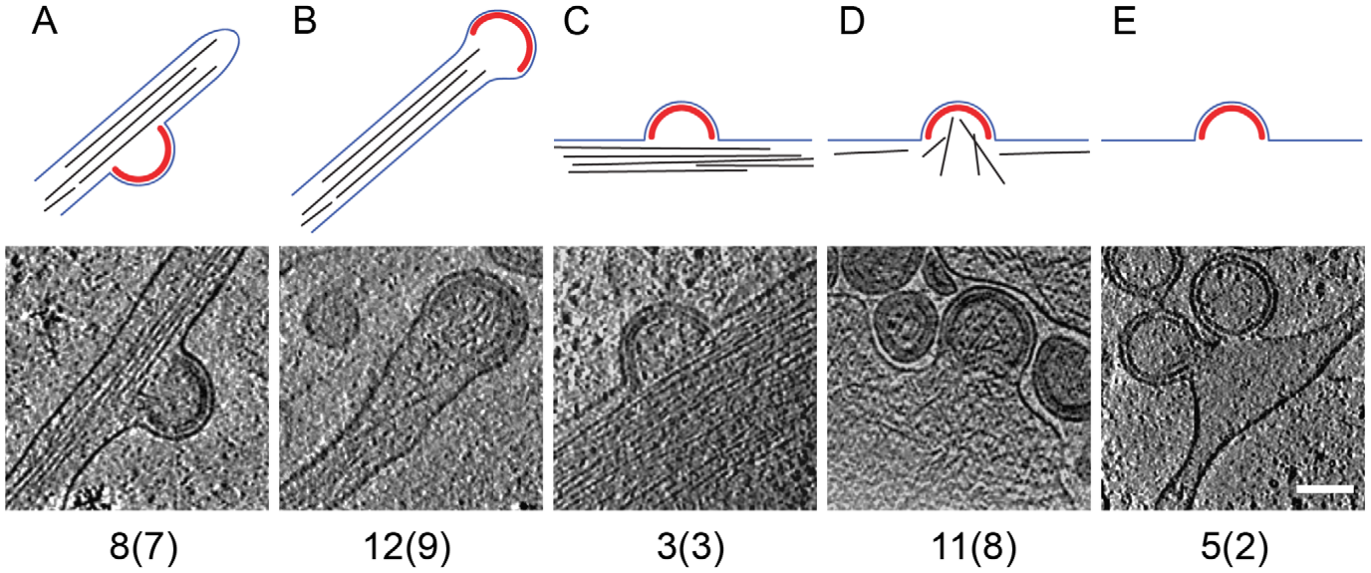
Filamentous actin at HIV-1 budding sites. The 39 budding sites reconstructed by cryo electron tomography were sorted into five categories, based on the type of filamentous actin structures (if any) present in their vicinity. Top panels are a sketch of the category, showing the Gag lattice in red, the plasma membrane in blue and filamentous actin in black. The lower panels show a computational slice through one budding site from the category, and the numbers below state the number of budding sites (and the number of tomograms they were obtained from) in the category. (A) Budding site at the side of actin-filled filopodium; (B) budding site at the tip of actin-filled filopodium; (C) budding site with cortical actin parallel to the plasma membrane; (D) budding site with cortical actin directed towards or protruding into the budding site; (E) budding site at the plasma membrane, not adjacent to filamentous actin. Scale bar is 100 nm.

In cryo electron tomograms of AdGag or AdGagPol transduced cells (Fig. 2) structural features of the cytoplasm were preserved at the high level previously reported for cellular cET [30], with single ribosomes, actin filaments and microtubules being clearly resolved. Both viral budding sites (Fig. 2A, C–F) and released virions (Fig. 2B–C, E) were present in the recorded tomograms. The budding sites were found at the plasma membrane on lamellipodia–like areas (Fig. 2C, E), occasionally in membrane invaginations (Fig. 2B, D) or at the tips (Fig. 2F, 3B) or sides (Fig. 2A, 3A) of filopodial structures. In the immature Gag lattice of these budding sites and released particles, the two density layers corresponding to CA and NC-RNA could be resolved, and occasionally the hexagonal symmetry in the CA layer was apparent by eye in more tangential tomographic slices. Released immature particles (ip) were found adjacent to cells expressing Gag (Fig. 2B, D) or GagPol (Fig. 2E), whereas released particles with mature morphology (mp) were only found adjacent to cells expressing GagPol (Fig. 2E). No density attributable to components of the ESCRT complex was detected at budding sites at the current resolution.

The preservation of cytoplasmic structure in cET allowed for three-dimensional snapshots of filamentous actin associated with HIV-1 budding sites. The presence of actin filaments was generally high at budding sites (Fig. 2A, C, F and Fig.3A–D), as judged by visual inspection where F-actin is clearly resolvable from *e.g.* intermediate filaments. Furthermore, we often observed what appeared to be a direct interaction of actin filaments with the budding site (Fig. 2A, F; Fig. 3D). To categorize the budding sites with respect to their actin context, they were sorted into 5 classes according to the type of actin structures they were associated with (Fig.3, top panel). This classification revealed that 34 of the 39 budding sites analyzed were found adjacent to filamentous actin (Fig. 3A–D), with half of the buds (20 of 39) appearing on the sides or tips of filopodia-like structures characterized by a parallel actin organization (Fig. 3A–B).

### *In situ* organization of the assembling Gag lattice

Cryo electron tomograms containing budding sites on intact cells with apparently high signal-to-noise ratio were selected for further analysis by sub-tomogram averaging as previously performed for isolated immature HIV-1 [19]. This analysis provides two kinds of information about the Gag protein lattice: the global arrangement of the repetitive elements into an ordered lattice structure (“lattice map”) and the local structure of these repetitive elements (“unit cell structure”).

**Figure 4:**
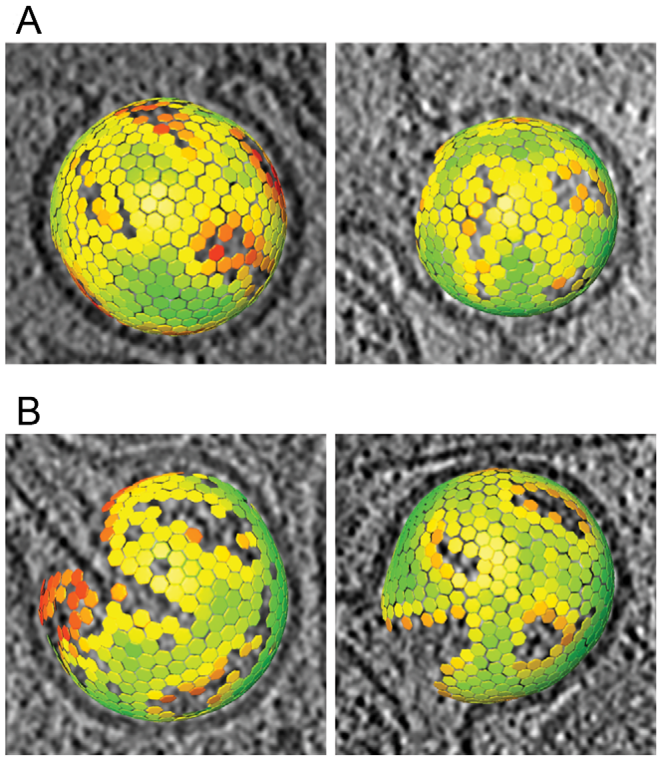
Lattice maps of immature and intermediate HIV-1 budding sites. Lattice maps of budding sites. The center and orientation of each aligned subtomogram is marked with a hexagon and is colored according to the cross correlation on a scale from low (red) to high (green). The lattice maps have the threshold set to the same value used for the subtomogram averaging procedure. The lattice map has been superimposed on a slice of 8 nm thickness through the budding viruses. (A) Lattice maps showing two budding particles with the newly reported lattice. (B) Lattice maps showing two budding particles with an immature lattice.

The immature Gag lattice parameters in the budding sites were the same as previously determined for released immature virions [5], [19]: a hexagonal lattice with a lattice constant of 8.0 nm. The lattice maps derived from the budding sites (Fig. 4A) revealed several sites of symmetry breakage, similarly heterogeneous in size and shape as those described for the released virions [19].

**Figure 5:**
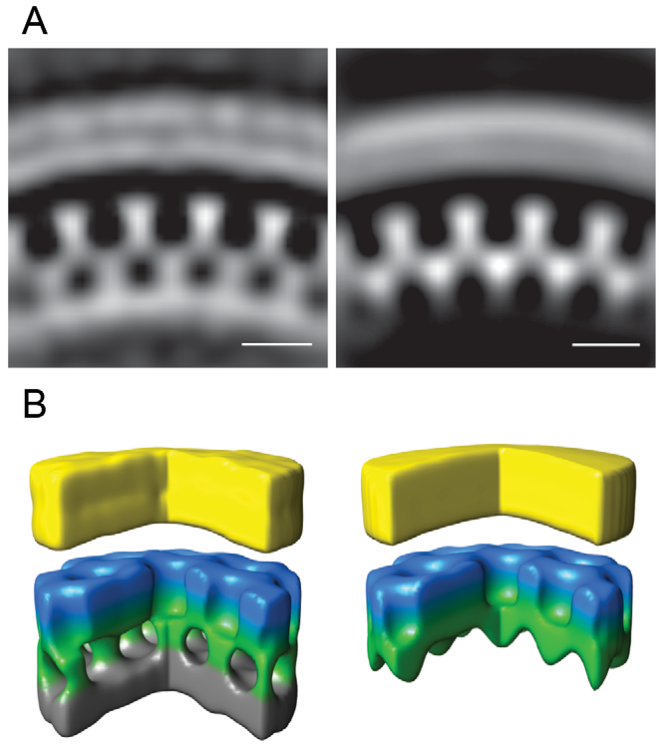
Gag unit cell structure in HIV-1 budding sites. Left panels show the average of the aligned subtomograms extracted from an individual budding site with the immature lattice. The right panels show the average from an individual budding site with the newly described lattice. (A) Central radial sections from the structures. Density is white. (B) Isosurface rendering of the structures. A segment of 90° has been cut out to better reveal the internal organization of the lattice. The surfaces have been colored radially to illustrate different domains in Gag: Yellow - membrane + MA; blue/green - CA; grey - NC + RNA. Scale bars 10 nm.

The unit cell structure derived from the cellular tomograms contained density corresponding to the N-terminal and C-terminal domains of CA, respectively, as well as density for the membrane and the NC-RNA layer (Fig. 5A). At the present resolution (roughly 40 Å by the 0.5 Fourier shell correlation criterion), this structure was indistinguishable from that previously reported for released particles [19].

### A previously undescribed Gag lattice form is present in tomograms of GagPol-expressing cells

**Figure 6:**
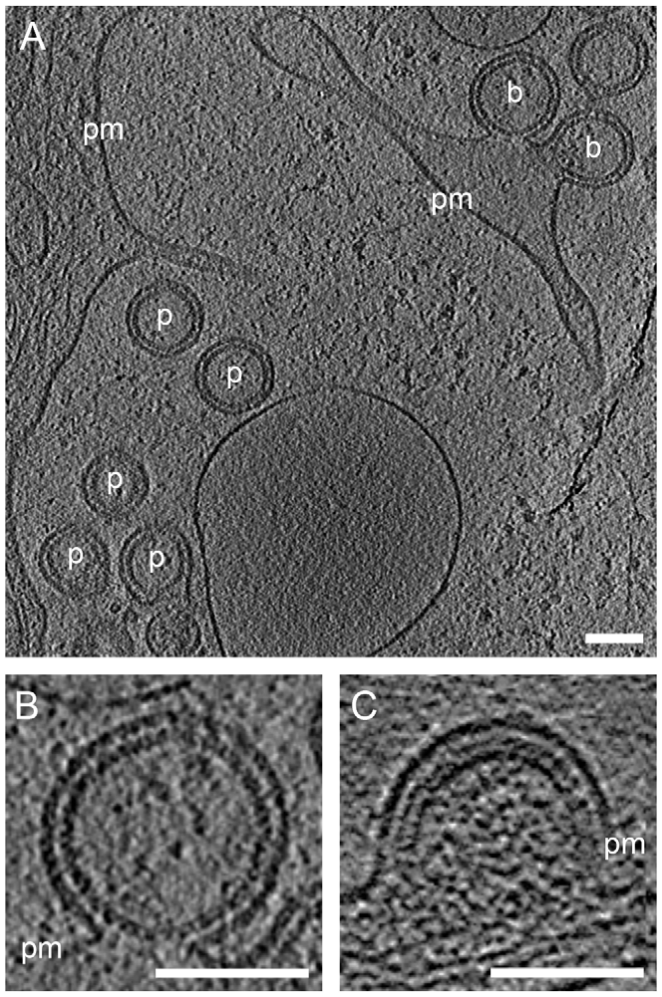
A previously undescribed Gag lattice in budding sites and released particles. (A) Computational slice through cryo electron tomograms of U-373 MG cells transduced with AdGagPol. Budding sites (b) and released particles (p) exhibit a Gag lattice different from both the immature and the mature lattice. (B) Enlarged slice through the top budding site from (A). Density is observed at the expected position of the CA domain, but no prominent density corresponding to NC-RNA is detected. (C) For comparison, a slice through a budding site with the immature Gag lattice showing the characteristic two density layers underneath the plasma membrane. Thickness of computational slices through cryo electron tomograms is 1.6 nm. pm: plasma membrane. Scale bars are 100 nm.

Immunoblot analysis of AdGagPol-transduced cells and the particle fraction recovered from the culture medium showed proteolytic processing of Gag (Fig. 1C). Furthermore, particles containing mature-looking cores were found in cryo electron tomograms in immediate proximity of cellular budding sites and next to immature particles (Fig. 2E). Of note, structures resembling the mature capsid were never found in budding sites still connected to the plasma membrane. Besides the well-described mature and immature particles, we observed a third, previously not described form of particles in the tomograms of AdGagPol-transduced cells (Fig. 6A). This apparently “intermediate morphology” exhibiting a thinner Gag lattice than the immature structure was observed both in extracellular particles (Fig. 6A, Fig. S2 in Supporting Information S1) and in HIV-1 budding sites connected to the cell (Fig. 6A–B). Budding sites and extracellular particles with the previously unrecognized Gag lattice were characterized by a single density layer bound to the inner face of the membrane (Figure 6B), as opposed to the two layers (CA and NC-RNA) seen in the immature lattice (Fig. 6C). Density plots orthogonal to the cell membrane confirmed that the density layer found in these novel structures was located at the same position relative to the plasma membrane (peak at −12 nm) as the CA layer of the complete immature lattice (Fig. S1 in Supporting Information S1).

Strikingly, budding sites and released particles with this novel Gag lattice displayed the same hexagonal symmetry and lattice spacing as the immature lattice (Fig. 4B). The apparently intermediate type lattices were typically more complete with smaller lattice defects than the immature lattices, but the lattice defects were similarly heterogeneous. Furthermore, the unit cell structure of the new lattice revealed an arrangement identical to the CA region of the immature lattice, with density corresponding to the N- and C-terminal domains of CA (Fig. 5B), but lacking any density corresponding to the NC-RNA layer. The cellular budding sites and extracellular particles containing this lattice also lacked the internal density corresponding to a condensed NC-RNA complex. Such densities were readily observed within mature capsids and in virions carrying partially processed Gag proteins (de Marco *et al.*, accompanying paper).

### Budding sites with the novel Gag lattice are also observed in infected T-cell lines

**Figure 7:**
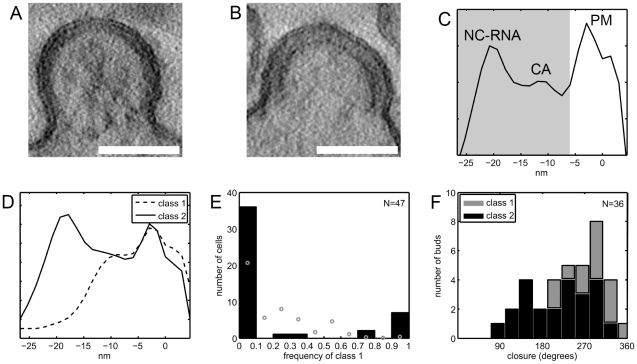
Budding sites with the novel lattice in HIV-1 infected T cells. (A–B) Computational slices of 1.5 nm thickness through tomograms of resin-embedded HIV-1 infected MT4 cells. By eye, two types of budding sites are discernible, one type having a thinner Gag density (A) and the other (B) the typical thickness of the immature Gag lattice. (C) Linear density profile of the Gag layer in one representative resin-embedded immature budding site. The profile was calculated orthogonally to the membrane in the central part of the budding site, as described in Materials and methods. Membrane center at 0 nm, negative direction corresponds to cell interior. Peaks are discernible at the position of the membrane leaflets (PM), the NC-RNA layer, and (less strong) the CA layer of the immature lattice. The shaded area indicates the part of the plots used for classification of bud types. (D) Budding sites in resin embeddings of infected T cells were classified based on line plots like the one shown in (C). The average plots for the two major classes differ at the position of the NC/RNA layer, class 1 (dashed line) resembling the novel lattice and class 2 (solid line) resembling the immature lattice. (E) Frequency of class 1 budding sites on 47 infected MT-4 T cells. Black bars indicate the number of cells carrying either no (frequency 0) or exclusively (frequency 1) class 1 budding sites or a mixture of class 1 and class 2 budding sites. The theoretical frequency distribution which would be expected if all cells exhibited the average frequency of 18% buds in class 1 is given by grey circles. This distribution has local maxima around 25% and 50% due to the limited number of budding sites sampled per cell, as explained further in the Supplementary Information. (F) Gag layer closure in budding sites. The previously published data on the closure of the Gag layer in budding sites on MT-4 cells show a broad distribution of morphologies [26]. Separating these data into the two classes of Gag densities shows that class 2 budding sites (black bars) are more similar to released immature HIV-1 particles, whereas class 1 budding sites (grey bars) exhibit a more closed Gag layer. Scale bars are 100 nm.

To determine whether the newly described lattice also occurs in other HIV-1 producing cells including T-cell lines, we recorded electron tomograms on sections of resin-embedded cells producing HIV-1. Although resin-embedded material is not preserved at the molecular level, it might still be possible to distinguish the two lattice types in these data, and this would allow recording sufficiently large data sets for statistical analyses. Visual inspection of resin-embedded sections from infected MT4 cells suggested the presence of a thinner density layer in some budding sites (Fig. 7A, B). This interpretation was validated based on the following criteria: (i) different types of membrane-bound densities in budding sites in resin embeddings could be classified by an unbiased scheme, and (ii) the presence of structures corresponding to the novel Gag lattice was dependent on an active viral PR.

Classification of budding sites and particles was based on linear density plots of the membrane-bound Gag density. Fig. 7C shows a typical example, demonstrating that enough structure is retained to resolve the plasma membrane and distinct regions in the Gag layer. The classification was based on the parts of the plots containing the density bound to the inner face of the membrane (shaded area in Fig. 7C). Data were recorded on resin embeddings of HIV-1 infected MT4 cells, HeLa cells transfected with a proviral HIV-1 plasmid as well as positive and negative controls for the presence of the two lattice types (Table 2). In order to avoid overrepresentation of single cells, two tilt series were recorded per cell, and they were recorded as far apart as possible in the section plane (usually >5 µm). The same cell was never studied in consecutive sections. Our published data on infected MT4 cells [26] were also included in the analysis (Table 2, data set 8).

**Table 2:** Data sets of resin embedded samples evaluated in the classification of Gag layer types.

Data set number	Sample	Number of tilt series	Number of cells in the data set	Number of budding sites or virions fully contained within sections
4	MT4 cells infected with HIV-1	85	50	224
5	MT4 cells infected with HIV-1 and cultured in presence of lopinavir	29	18	53
6	HEK293T cells transfected with pNL4-3-MACASP1 (only released virions)	7	-	16
7	HeLa cells transfected with wt pNL4-3	33	20	69
8	MT4 cells infected with HIV-1	19	-	36

The number of tilt series recorded, and containing budding sites or released particles, are stated for each data set. Data set 8 was previously evaluated for Gag layer closure [26], whereas data sets 4–7 were recorded in a manner to allow for statistical evaluation of frequencies of Gag lattice types (see text).

Principal component analysis of the variance in the data set revealed the presence of one major cluster with residual variance and a smaller, more homogeneous one (Fig. S3A–D in Supporting Information S1). Hierarchical clustering of the cropped line plots identified the two main classes as the two clusters observed in the PCA representation (Fig. S3E,F in Supporting Information S1). The less populated class (class 1, dashed line in Fig. 7D) lacked the innermost density peak corresponding to NC-RNA, whereas this peak was present in the more populated class (class 2, solid line in Fig. 7D). Further division of class 2 into subclasses mainly revealed a slight variation in the position and relative intensity of the NC-RNA peak (Fig. S4 in Supporting Information S1).

Table 3 shows the distribution of budding sites into the two classes for each sample. Class 1 contained 18% of the budding sites on HIV-1 infected MT4 cells and 2.9% of the budding sites on transfected HeLa cells. For infected MT4 cells treated with the PR inhibitor lopinavir, no class 1 budding sites were observed. As a control for the thinner Gag lattice, released virions carrying uncleaved MA-SP1 were studied. In this case, Gag processing is stalled at the MACASP1 fragment due to inactivating mutations at cleavage sites between MA and CA and CA and SP1, respectively (see [10] and accompanying paper by de Marco *et al.*). These particles generally fell into class 1. Taken together, these results suggest that class 1 corresponds to the newly described lattice detected by cET, while class 2 corresponds to the immature lattice.

**Table 3:** Number of budding sites or virions assigned to class 1 and class 2 Gag layer morphology, respectively, for the different sample groups.

Sample (data set number)	Number of budding sites or virions	Class 1	Class 2	Frequency of class 1 (%)	Frequency of class 1, 95% confidence interval (%)
MT4 cells infected with HIV-1 (4)	224	41	183	18	13.5–24.0
MT4 cells infected with HIV-1 in the presence of lopinavir (5)	53	0	53	0	0.0–6.7
Particles containing uncleaved MA-SP1 (6)	16	15	1	-	-
HeLa cells transfected with wt pNL4-3 (7)	69	2	67	2.9	0.35–10
MT4 cells infected with HIV-1 (8)	36	13	23	-	-

Where applicable (data acquisition scheme allowing for statistical interpretation), a frequency and its 95% confidence interval was calculated using the MATLAB function *binofit*, which uses an F-function approximation of the exact Clopper-Pearson formula [41].

Radius and closure of the Gag layer was calculated separately for the two classes (Table 4, Fig. 7F). Whereas the average radii of the two classes were similar, the closure of the Gag layer was significantly higher for the class 1 buds (Wilcoxon rank sum test, p = 0.0021). Assuming the same lattice packing in both classes, class 1 budding sites in this data set contain 5,000±1,300 Gag molecules while class 2 budding sites in this data set contain 3,300±1,500 Gag molecules.

**Table 4:** Properties of budding sites on MT4 cells according to Gag layer class.

	Class 1	Class 2	Total
Number of buds	13	23	36
Radius (nm)	76±5	74±8	75±7
Closure (°)	289±45	218±70	244±70
Number of Gag molecules per bud	5,000±1,300	3,300±1,500	3,900±1,600

The calculation is based on the published evaluation of data set 8 (Table 2), assuming the same Gag packing density in both class 1 and class 2, which is valid if they represent the NC-RNA-less and immature lattice, respectively.

Finally, we asked whether individual cells carried only one class of budding sites or both. In the case of HIV-1 infected MT4 cells, 47 cells exhibited more than one budding site in the tomogram (Table 2, data set 4). Fig. 7E (black bars) shows the relative frequency of class 1 budding sites on these 47 cells, overlayed with the theoretical distribution (grey circles) that would be expected if all cells had the average of 18% class 1 budding sites. Clearly, most cells had either only class 1 or only class 2 budding sites, indicating that the budding phenotype is determined at the single cell level.

## Discussion

Here, we established a system to visualize HIV-1 virus-like particle assembly and release *in situ* at macromolecular resolution in three dimensions using cET. This method permits structural interpretation of assembly sites and their cytoplasmic and membrane surroundings without the limitations imposed by the fixation, staining and dehydration methods commonly used in cellular electron microscopy. It allowed a detailed analysis of the Gag lattice in cellular budding sites, suggesting that the organization of the immature HIV-1 particle is already determined at the point of its intracellular assembly, and further identified a hitherto undescribed Gag lattice form. We show that this Gag lattice form is predominant in a subset of infected T-cells, and hypothesize that it is the result of premature PR activation in these cells.

The cET analysis of HIV-1 Gag and Gag-Pol expressing cells also provided unprecedented snapshots of cortical actin filaments in the vicinity of viral budding sites (Fig. 2, 3). It is well established that HIV-1 particles contain substantial amounts of actin [31], but the interplay between cortical actin and retrovirus assembly is still unknown. A recent study indicated a role for star-shaped actin filament arrangements emanating from the viral budding sites in HIV-1 assembly [32]. Our cET data provide 3D structures suggesting directed arrangement of actin filaments towards some budding sites, particularly in filopodia-associated buds. Strikingly, half of the budding sites in the current data set were present on actin-filled filopodia. To what extent this is a consequence of the particular region of the cell accessible to cET remains to be determined. Future cET studies using altered Gag constructs and selective manipulation of the actin dynamics will provide the necessary tools to untangle the molecular details of the involvement of actin at HIV-1 budding sites.

Extracellular immature HIV-1 particles are the direct products of viral budding at the plasma membrane, making it likely that their Gag protein lattice is determined during assembly. The observation that the Gag lattice is incomplete in the immature virus [22], [26] and contains defects [19], [22] raised the alternative hypothesis that Gag rearrangements occur following release. This could involve post-release association of smaller patches of Gag hexamers, or post-release symmetry breakage and dissociation of a hexagonal lattice containing evenly spaced pentameric defects [22]. Analysis of the lattice in extracellular immature virions revealed continuous hexameric symmetry over most of the lattice with curvature induced by defects of irregular size and shape [19]. These studies were performed on purified released particles, however, and the degree of order attained during assembly at the plasma membrane may have been higher (and broken after release) or lower (and subsequently assembled into a more ordered lattice). Our cET data of budding sites revealed structures of the immature Gag lattice in intact unperturbed cells prior to release, allowing us to draw conclusions on the early steps of the HIV-1 assembly process. There was no indication of separated “islands” of hexameric Gag in the *in situ* budding sites; upon visual inspection they consistently contained one continuous layer of Gag protein. A more detailed analysis of the lattice in the immature budding sites revealed that its unit cell structure was highly similar to that previously described for released immature virions [19], and included irregular lattice defects of similar size and distribution. This suggests that the organization of the immature HIV-1 particle is indeed determined at the point of its intracellular assembly, and not as a result of large scale post-release ordering or disordering.

Besides the described immature Gag lattice, we observed a previously undescribed lattice type in extracellular particles and in budding sites still connected to the cell. Formation of this lattice was dependent on an active HIV-1 PR. The newly discovered lattice exhibited hexagonal symmetry with a CA layer identical to the immature lattice, but lacked the second layer of density corresponding to the NC-RNA complex. The particles and budding sites also contained no visible NC-RNA condensate in the center of the particles or adjacent to the buds, suggesting that the viral genome is absent. A comparison with the analysis performed in the accompanying paper by de Marco *et al.* on isolated virions with specific Gag cleavage site mutations showed that the newly described lattice is most likely composed of the MA-SP1 region of HIV-1 Gag. Although the tomographic average at current resolution does not allow identification of the density corresponding to the SP1 peptide, de Marco *et al.* showed that this peptide is present, independent of whether the cleavage site between CA and SP1 is intact or not. Notably, the particles produced from the MA-CA and MA-SP1 cleavage site mutants which exhibit an MA-SP1 type lattice do contain a clearly distinguishable density corresponding to condensed NC-RNA (de Marco *et al.*), which distinguishes them from the particles with this lattice type in the current study.

As further described in the accompanying paper by de Marco *et al.*, the MACASP1 lattice is likely to constitute a natural intermediate in the proteolytic maturation of HIV-1. Cleavage between SP1 and NC is the fastest processing step *in vitro* and abscission of the C-terminal NC-p6 regions from the immature lattice is required for condensation of the inner ribonucleoprotein complex of the virion prior to formation of the capsid. Thus, extracellular particles containing the MACASP1 lattice and inner density corresponding to NC-RNA are suggested to represent a “frozen” intermediate in HIV-1 maturation (de Marco *et al.*). Such particles were not observed for wild-type HIV-1 constructs, however, indicating that maturation intermediates are normally short-lived. An MACASP1 lattice without adjacent NC-RNA density was observed in 18% of HIV-1 budding sites on infected MT-4 cells, on the other hand, and its frequent presence suggests that it is a metastable structure with a significant lifetime in this case.

We previously reported that budding sites on infected MT4 cells have a broad distribution of Gag layer morphologies. Some resemble actual released immature virions, but some have a more complete Gag layer, containing more Gag molecules than the released virions and thus rather resemble arrested “late” budding sites not thought to be virus precursors [26]. When re-evaluating these data with respect to the type of Gag lattice, the immature lattice was predominantly found in budding sites resembling released virions, whereas the budding sites with the novel lattice had a significantly more closed Gag layer than released virions (Table 4, Fig. 7F). In the case of late-domain defective variants, formation of a closed layer is thought to be caused by a failure to recruit the ESCRT machinery and thus to drive the release process. Consequently, Gag assembly continues until reaching an equilibrium structure, but the resulting late budding sites are mostly dead-end products. It appears likely that a similar process is also relevant for the budding sites with the novel lattice. We hypothesize that premature proteolytic activation prior to confinement of virion constituents in the budding virion leads to removal of NC-p6 from Gag and of PR and downstream *pol* products from Gag-Pol. The cleaved C-terminal fragments are lost from the assembly site, while the MACASP1 lattice is stably retained. No ESCRT recruitment occurs since the p6 domain has been removed and no further processing occurs because PR, not being confined in a viral particle, has been lost. Accordingly, no budding structures with mature cores were observed. Further assembly of the truncated lattice may involve additional Gag molecules undergoing partial processing or addition of cytoplasmic MACASP1 molecules since a truncated MACASP1 construct has been shown to form budlike structures when overexpressed [33], [34]. The presence of extracellular particles with the MACASP1 lattice and lacking NC-RNA may indicate some ESCRT-independent release. Release of virions lacking most of the NC domain (but in this case carrying the p6 domain) has also been observed in previous studies [35], [36].

The results of the current study clearly show that morphological maturation can be initiated inside the producer cell, but indicate that it is largely non-productive if the virion constituents are not confined in a structure sequestered from the cytoplasm. Assembly, release and maturation would therefore require the sequential processes of Gag association, ESCRT recruitment (prior to closure of the Gag shell), and PR activation (after or concomitant with ESCRT-mediated sequestration of the bud). The observation of aberrant, apparently dead-end structures in a subset of cells in HIV-1 infected T-cell lines suggests that the control of these processes can be lost, however. Infected MT-4 cells generally contained only one type of budding sites indicating that this loss of control may be caused by the host cell environment. Conceivably, host cell factors may be involved in regulating the kinetics of the consecutive stages of viral assembly, budding and release, and the subset of T-cells may have lost this regulation, although a general cytopathic effect cannot be ruled out at present. Identifying viral or host factors involved in this regulation will be of great importance for our understanding of HIV-1 morphogenesis, and may also lead to new approaches to render HIV-1 infection non-productive.

## Materials and Methods

### Construction of adenoviral vectors for HIV-1 Gag and GagPol

Adenoviral vectors for HIV-1 Gag (AdGag) and GagPol (AdGagPol) were constructed from the Rev independent versions of these genes [29], using the BD Adeno-X Expression System 1 (BD Biosciences Clontech). Briefly, the 1539 nt *gag*-gene was amplified by PCR adding flanking *NheI/XbaI* sites and ligated into the transfer vector pShuttle2. The *gag-pol* gene was excised with *NdeI/XbaI* from pcDNA3.1syngagpol [29] and directly cloned into pShuttle2. After verification of the insert by sequence analysis, these plasmids were digested with *PI-Sce/I-Ceu*. The inserts were ligated into the pre-digested Adeno.X viral DNA. Resulting plasmids were verified by restriction digest and DNA sequencing. Recombinant adenoviruses were generated by transfection of the AdGag or Ad GagPol plasmids into HEK 293 cells and amplified according to the protocol supplied with the kit. Titers of adenoviral vectors were determined using the Adeno-X rapid Titer Kit (Clontech); titers of both AdGag and Ad GagPol typically reached 1–2×10^9^/ml. Immunofluorescence staining of AdGag and AdGagPol transduced cells for HIV-1 Gag was performed using a rabbit polyclonal anti-CA antibody and a FITC-conjugated goat-anti-rabbit secondary antibody. Western blots of cell lysates and particles purified from the culture media of AdGag and AdGagPol transduced cells were performed using the same polyclonal anti-CA antibody, with an IRDye 800CW goat anti-rabbit secondary antibody (LI-COR Biosciences) as recommended by the manufacturer.

### Cell culture and adenoviral vector transductions for cryo electron tomography

Human glioblastoma cell lines U-87 MG and U-373 MG were maintained in DMEM and MEM, respectively, supplemented with Penicillin-Streptomycin and 10% foetal bovine serum. When grown on EM grids (CF-2/1-2AU, Protochips, inc.), cells were seeded at low density and kept in medium with 0.5% serum which increased the extent of flat areas of the cells. Transduction with AdGag or AdGagPol was performed concomitant with or one day after seeding cells on EM grids. A multiplicity of ‘infection’ (MOI) of 10 was typically used, calculated based on cell culture dish area and assuming that added vector will adhere to any substrate surface. One to three days after seeding cells on the grids, they were plunge-frozen into liquid ethane after application of 10 nm colloidal gold.

### Sample preparation for electron tomography of sections of resin-embedded cells

Cell culture and preparation for sections of resin embedded cells were performed as previously described [26]. For production of immature virus, cells were grown in the presence of 1 µM lopinavir. For production of particles containing an uncleaved MA-SP1 layer, HEK293T cells were transfected with a proviral HIV-1_NL4-3_ plasmid carrying mutations in the cleavage sites between MA and CA and CA and SP1, respectively ([14] and accompanying paper by de Marco *et al.*). Cells were fixed, stained, dehydrated and EPON-embedded, and 300 nm sections of the embeddings were cut and transferred to EM grids after deposition of colloidal gold onto the EM grid support film.

### Electron tomography

Cryo electron tomography was performed with a Philips CM300 or a FEI Tecnai F30 Polara transmission electron microscope (FEI; Eindhoven, The Netherlands), both equipped with 300 kV field emission guns, Gatan GIF 2002 post-column energy filters and 2048×2048 Multiscan CCD cameras (Gatan; Pleasanton, CA). All data collection was performed at 300 kV, with the energy filter operated in zero loss mode. Tilt series of cryo specimens were typically recorded from −60° to +60° with an angular increment of 1.5°, a total electron dose of 80 electrons Å^−2^, and a defocus of −6.0 µm to −8.0 µm. The pixel sizes at the specimen level were 8.21 Å at the CM300, and 8.05 Å or 7.13 Å at the FEI Tecnai F30 Polara, respectively. Electron tomography of resin sections was performed at an FEI Tecnai F20 transmission electron microscope (FEI; Eindhoven, The Netherlands), equipped with 200 kV field emission gun and a 4096×4096 Eagle CCD camera (FEI; Eindhoven, The Netherlands). Tilt series on resin sections were recorded at room temperature from −60° to +60° with an angular increment of 1.0°, with a one time binned pixel size of 2*3.72 Å and a defocus of -4.0 µm. For both cryo and room temperature tilt series, the applied electron dose for a given tilt angle α was proportional to 1/cos(α) to compensate for the higher effective specimen thickness at higher tilts. At the Tecnai microscopes, the SerialEM acquisition software [37] was used for tilt series acquisition. Three-dimensional reconstructions from tilt series were calculated using the weighted back-projection method [38], as implemented in the TOM toolbox [39] for MATLAB (Mathworks, Natick, Massachusetts, United States) with the “exact weighting” option, or in IMOD [40].

### Subtomogram averaging

Subtomogram averaging was performed using MATLAB. Sub-volumes of (38.6 nm)^3^ were extracted along the surface of the budding particles. The subtomograms were then iteratively aligned against the average in a reference free manner, while applying 6-fold symmetry as described in the accompanying article (de Marco *et al*). For individual particles, the distribution of the cross correlation values between the aligned subtomograms and the reference was bimodal. The threshold of the subtomograms that went into the reconstruction was set as minimum between the two peaks.

### Classification of Gag lattice types in tomograms of resin-embedded sections

The classification of Gag densities in resin embeddings of HIV-1 budding sites was performed as described in the Supplementary Information. In brief, linear density profiles were calculated orthogonally to the plasma membrane in the budding sites. These one-dimensional density profiles were cropped to contain only the membrane-apposed density, and subjected to hierarchical clustering.

## Supporting Information

Supporting Information S1 Supporting image analysis procedures and additional cryo electron tomography images.
(1.45 MB PDF)
