# Supplementary material for: Cryo Electron Tomography of Native HIV-1 Budding Sites
Source: PLoS Pathog. 2010 Nov 24;6(11):e1001173. doi: 10.1371/journal.ppat.1001173 (PMC2991257; doi:10.1371/journal.ppat.1001173)
Supplement: Supporting Information S1 — Supporting image analysis procedures and additional cryo electron tomography images. (1.45 MB PDF) [file ppat.1001173.s001.pdf]

Supplementary Information

Cryo Electron Tomography of native HIV-1 Budding Sites

Lars-Anders Carlson[1,2], Alex de Marco[3], Heike Oberwinkler[1], Anja Habermann[1], John A.G. Briggs[3], Hans-Georg Kräusslich[1,*], Kay Grünewald[2,4*]

[1]Department of Infectious Diseases, Virology, Universitätsklinikum Heidelberg, Im Neuenheimer Feld 324, D-69120 Heidelberg, Germany

[2]Dept. Molecular Structural Biology, Max-Planck-Institute of Biochemistry, Am Klopferspitz 18, D-82152 Martinsried, Germany

[3]Structural and Computational Biology Unit, European Molecular Biology Laboratory, Meyerhofstrasse 1, 69117 Heidelberg, Germany

[4]Oxford Particle Imaging Centre, Division of Structural Biology, Wellcome Trust Centre for Human Genetics, University of Oxford, Roosevelt Drive, Oxford OX3 7BN, UK

*joint corresponding authors

HGK: Tel +496221 565001; Fax +496221 565003;

Email: hans-georg.kraeusslich@med.uni-heidelberg.de

KG: Tel +441865 287817; Fax +441865 287547;

Email kay@strubi.ox.ac.uk

Classification of Gag lattice types in tomograms of resin-embedded sections

The position of the plasma membrane in the budding sites was manually traced using IMOD [1] on every third xy-slice in the central 30 slices of the budding sites (where the membrane was cut in an angle of 90±~20° by the tomographic slice). From this set of linearly connected points, a triangular mesh surface of the plasma membrane was interpolated and smoothed using the IMOD functions smoothsurf, imodmesh and imodfillin, combined with custom-made scripts for MATLAB (Mathworks, Natick, Massachusetts, United States). The points in this surface model have an approximate spacing of one voxel, and the surface normal at every point can be calculated using the connection to the neighboring points. Using this, the tomogram grey values along a line orthogonal to the membrane were calculated for every point, and the average of these one-dimensional plots over the membrane surface was calculated.

When necessary, the resulting line plots were scaled to achieve the same object pixel size. They were cropped to either [-27 nm, -6 nm] or [-27 nm, 4.5 nm] (where 0 nm is the center of the membrane and the negative direction is the cell/virion interior) to contain either the membrane-bound density or the membrane-bound density and the membrane. After normalization of the scaled and cropped line plots to average 0 and standard deviation 1, they were subjected to further analysis.

Classification of the line plots was performed in MATLAB using functions in the statistics toolbox. Principal component analysis [2] was performed with the MATLAB function *princomp*, hierarchical clustering with the MATLAB functions *pdist*, *linkage* and *cluster*. Briefly, the distance between the 15 element vectors (containing the membrane-bound density) was calculated as the Euclidean distance and was used for linkage analysis with *linkage*, using

Ward's linkage criterion of increase in intragroup variance. Linkage analysis using the criterion of mean intercluster distance gave indistinguishable results.

Calculation of the theoretical frequency distribution in Figure 7E

The gray circles in Figure 7E represent the theoretical frequency distribution expected if all cells had 18% budding sites in class 1 (thin lattice). This distribution was calculated by taking into account the small number of budding sites sampled per cell, in the following way:

(i) If the same number n of budding sites had been recorded on every cell, the probability of having k ($k \leq n$) budding sites in class 1 (thin lattice) is given by the Binomial distribution

$$P_n(k) = (n!/(k!(n-k)!)) \times 0.18^k \times (1-0.18)^{(n-k)}.$$

The frequency (x-axis in Figure 7E) is given by k/n and its probability (y-axis in Figure 7E) is given by $P_n(k)$.

(ii) The actual experimental data contain a varying number of budding sites per cell. We denote the probability of n budding sites on a cell in the data set by W_n. The theoretical frequency distribution displayed in Figure 7E is then given as the weighted average of the $P_n(k)$ by W_n, where the frequencies k/n for each individual $P_n(k)$ was rounded to the closest 0.1 for the histogram representation. This combined frequency distribution has local maxima around 0.25 and 0.5 since these values are among the few that can be formed for certain values of n (2,4,8) that occur often in the data.

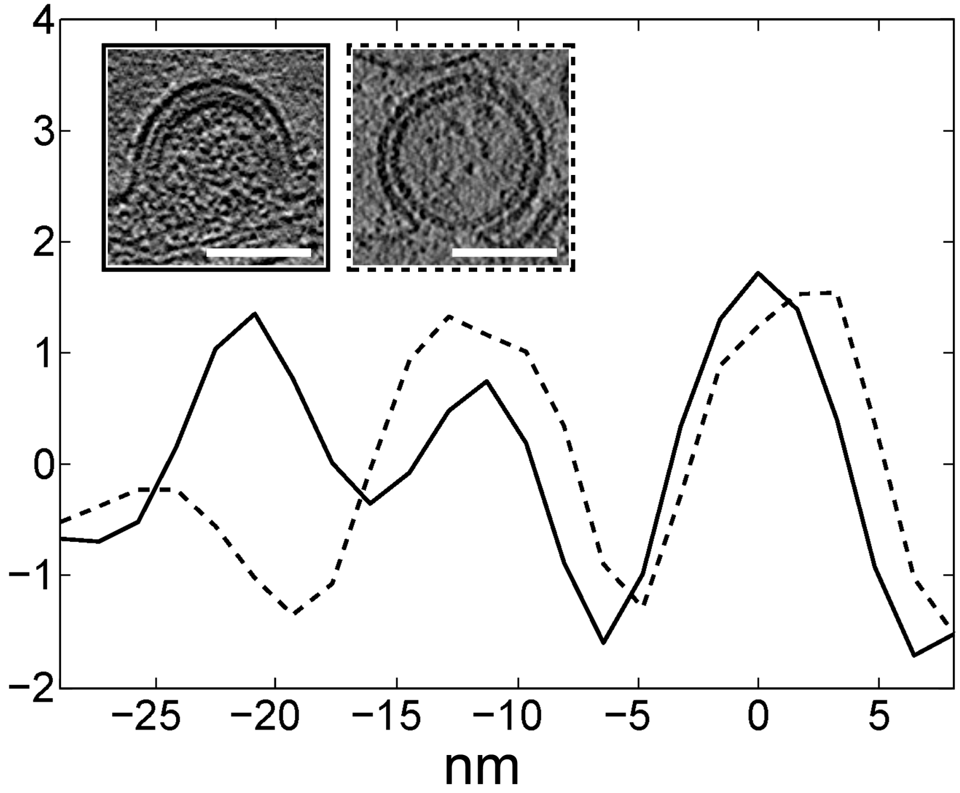

Figure S1. Comparison of budding sites with the immature or the newly described thinner lattice in cryo electron tomograms. Linear density profiles were produced by integration of the tomogram density perpendicular to the plasma membrane in one budding site with the immature (solid line) and one with the newly described (dashed line) lattice. Membrane at 0 nm, negative direction corresponds to cell interior. The three peaks in the immature budding site correspond to the plasma membrane and the known positions of the CA and NC/RNA layers of the immature Gag lattice. The budding site with the newly described lattice has the CA peak at the same distance as the immature budding site, but lacks the NC/RNA peak. The inset show computational slices of 1.6 nm thickness through the cryo electron tomograms of the analyzed budding sites with the immature (left) and newly described (right) lattice. Scale bars are 100 nm.

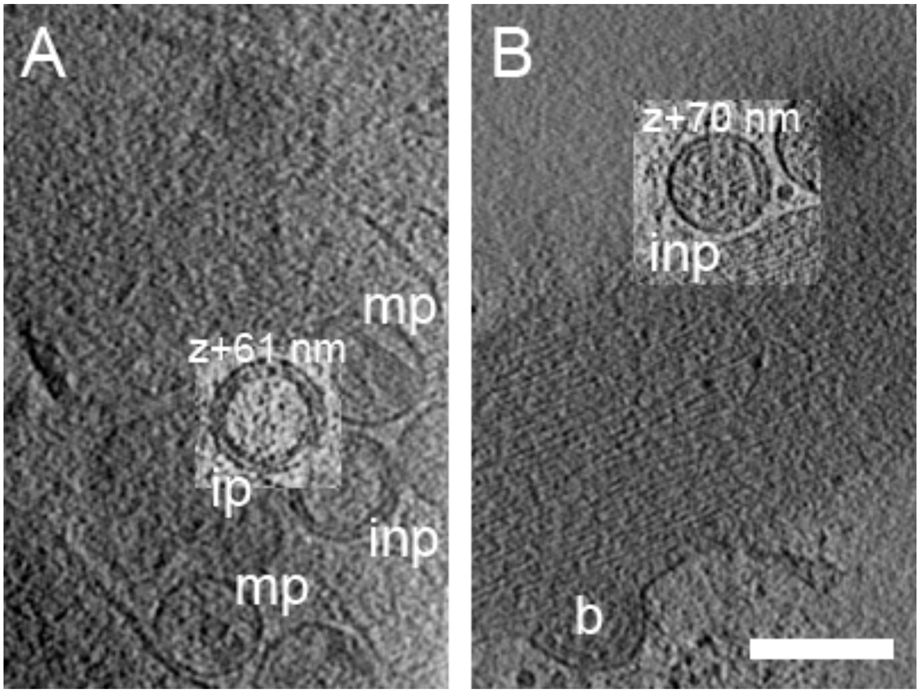

Figure S2. The newly described Gag lattice in released particles. Computational slices of 3.2 nm thickness through cryo electron tomograms of cells infected with AdGagPol. Released particles with the newly described lattice were found next to released particles with immature and mature morphology (A), as well as adjacent to budding sites with an immature Gag layer (B). Structure designation: b, budding site, ip, immature particle, inp= particle with an apparently intermediate lattice, mp=mature particle. Scale bar is 200 nm.

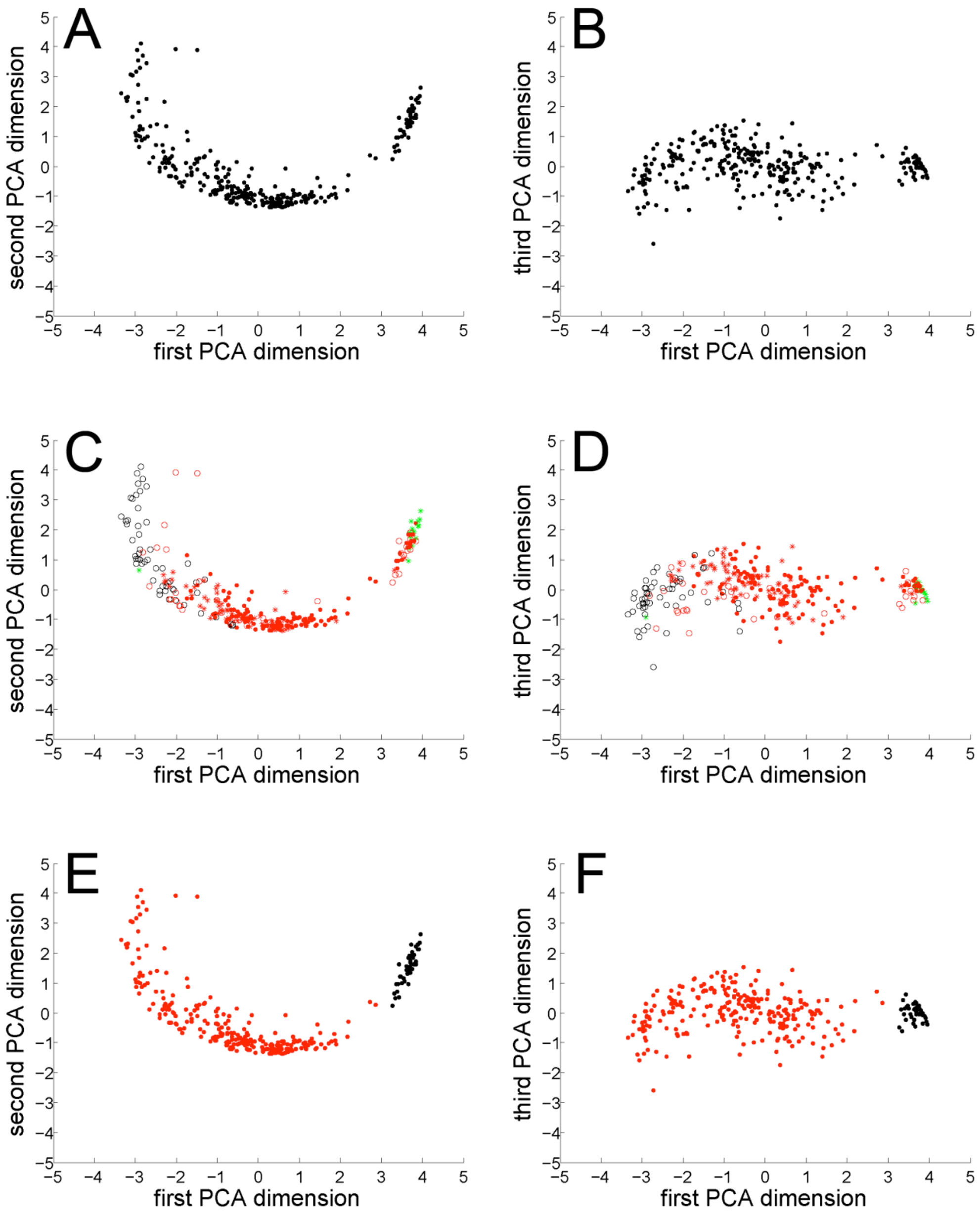

Figure S3. Visualization of variance between budding sites by principal component analysis (PCA). The cropped density profiles were subjected to PCA, and projected on the first and second (A, C, E) or the first and third (B, D, F) base vectors, respectively. (A-B) All data points. (C-D) All data points, colored according to sample group listed in Table 2: MT4 co-culture (data set 4), filled red circles, MT4 co-culture (data set 8), open red circles; HeLa wt, red stars; MT4 co-culture+lopinavir, open black circles; and released MACASP1 virions, filled green circles. (E-F) All data points, colored according to the class assignment in the hierarchical classification, class 1 in black, class 2 in red.

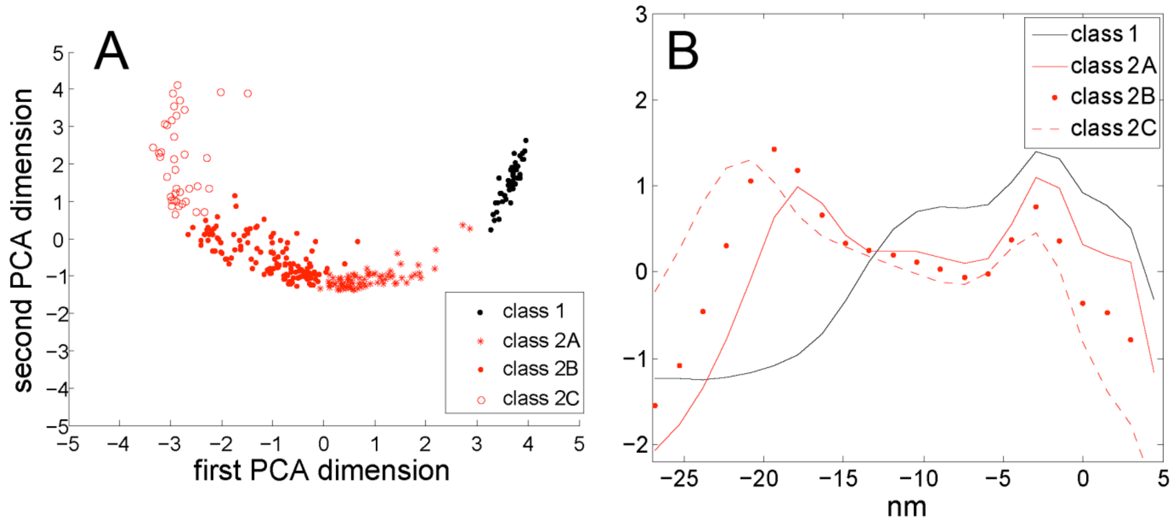

Figure S4. Variance within class two is mainly NC-RNA position. When four instead of two classes are separated from the hierarchical clustering (A), the main variance in the more populated class 2 of Figure S3 is seen to be slight variations in the position of the NC-RNA layer (B). There are also variations in the membrane region between the four classes, but since this part of the density profile is not used in the PCA or clustering (but only the shaded part as indicated in Fig. 6C), it will not contribute to the variation in class 2 found there.

References

1. Kremer JR, Mastronarde DN, McIntosh JR (1996) Computer visualization of three-dimensional image data using IMOD. Journal of Structural Biology 116: 71-76.
2. Duda RO, Hart PE, Stork DG (2001) Pattern Recognition. New York: John Wiley & Sons, Inc.
